# Isolation and characterization of a tandem-repeated cysteine protease from the symbiotic dinoflagellate *Symbiodinium* sp. KB8

**DOI:** 10.1371/journal.pone.0211534

**Published:** 2019-01-31

**Authors:** Yuya Suzuki, Tomohiro Suzuki, Koichiro Awai, Yuzo Shioi

**Affiliations:** 1 Graduate School of Science, Shizuoka University, Shizuoka, Japan; 2 Research Institute of Green Science and Technology, Shizuoka University, Shizuoka, Japan; 3 Research Institute of Electronics, Shizuoka University, Hamamatsu, Japan; 4 PRESTO, JST, Kawaguchi, Japan; Shantou University Medical College, CHINA

## Abstract

A cysteine protease belonging to peptidase C1A superfamily from the eukaryotic, symbiotic dinoflagellate, *Symbiodinium* sp. strain KB8, was characterized. The protease was purified to near homogeneity (566-fold) by (NH_4_)_2_SO_4_ fractionation, ultrafiltration, and column chromatography using a fluorescent peptide, butyloxycarbonyl-Val-Leu-Lys-4-methylcoumaryl-7-amide (Boc-VLK-MCA), as a substrate for assay purposes. The enzyme was termed VLKP (VLK protease), and its activity was strongly inhibited by cysteine protease inhibitors and activated by reducing agents. Based on the results for the amino acid sequence determined by liquid chromatography–coupled tandem mass spectrometry, a cDNA encoding VLKP was synthesized. VLKP was classified into the peptidase C1A superfamily of cysteine proteases (C1AP). The predicted amino acid sequence of VLKP indicated a tandem array of highly conserved precursors of C1AP with a molecular mass of approximately 71 kDa. The results of gel-filtration chromatography and SDS-PAGE suggested that VLKP exists as a monomer of 31–32 kDa, indicating that the tandem array is likely divided into two mass-equivalent halves that undergo equivalent posttranslational modifications. The VLKP precursor contains an inhibitor prodomain that might become activated after acidic autoprocessing at approximately pH 4. Both purified and recombinant VLKPs had a similar substrate specificity and kinetic parameters for common C1AP substrates. Most C1APs reside in acidic organelles such as the vacuole and lysosomes, and indeed VLKP was most active at pH 4.5. Since VLKP exhibited maximum activity during the late logarithmic growth phase, these attributes suggest that, VLKP is involved in the metabolism of proteins in acidic organelles.

## Introduction

*Symbiodinium* species are eukaryotic, photosynthetic dinoflagellate algae that produce the light-harvesting carotenoid, peridinin. Although they can assume free-living forms with flagella, they usually reside in the endodermis of tropical invertebrates, e.g., corals, giant clams, jellyfish, and sea anemones. Their symbiotic relationship with corals and these other organisms allows corals to use the algal photosynthetic products for >90% of the energy required to maintain their homeostasis, growth, and calcification [1], whereas *Symbiodinium* species use host metabolites, e.g., carbon dioxide, ammonia, urea, and amino acids [2, 3]. Corals take advantage of the symbiosis to form hard, calcium carbonate skeletons that form the structural basis for reefs in otherwise oligotrophic tropical seas.

Certain cysteine proteases (CPs), i.e., those for which activity is dependent on an active-site cysteine, are involved in maintaining symbiotic relationships. The pea aphid *Acyrthosiphon pisum* harbors the enterobacterium *Buchnera* and coordinates *Buchnera* density with its growth stage via the *A*. *pisum* CP, cathepsin L-like protease [4]. The ciliate parasite *Philasterides dicentrarchi* also uses a cathepsin L–like protease to attack host fish [5]. The malaria protozoan *Plasmodium falciparum*, an apicomplexa, invades host erythrocytes with use of the CP, falcipain [6]. A CP, CysP of the pathogenic bacterium *Mycoplasma*, can cleave chicken IgG into its Fab and Fc fragments [7]. Given that CPs are involved in symbiosis, we hypothesized that a *Symbiodinium* CP(s) might exist and play a role in symbiosis. Furthermore, although genomic and transcriptomic studies of algal CPs have been performed [8, 9], little direct information is available for these enzymes.

For the study reported herein, we characterized the physical and biochemical properties of a CP from *Symbiodinium* sp. KB8, which had been isolated from the upside-down jellyfish (*Cassiopea* sp.) [10]. Among six fluorescing peptide substrates tested, proteolytic activity in a crude *Symbiodinium* sp. KB8 extract was greatest for butyloxycarbonyl-Val-Leu-Lys-4-methylcoumaryl-7-amide (Boc-VLK-MCA). Although Boc-VLK-MCA is a known substrate for plasmin and calpain, which are not found in photosynthetic organisms, it has been shown to be degraded by some CPs [11]. Therefore, we named the enzyme associated with this activity VLK protease (VLKP). In addition to purifying and biochemically characterizing VLKP, we sequenced its gene, produced recombinant VLKP (rVLKP) in *Escherichia coli*, and compared the substrate specificities of native VLKP and rVLKP. Based on our results, we propose a possible physiological function(s) for VLKP.

## Materials and methods

### *Symbiodinium* sp. KB8 culture

*Symbiodinium* sp. KB8 algal cells isolated from the upside-down jellyfish were cultured in 3 l of f/2 medium [12] under 40–80 μmol photon m^−2^ s^−1^ light at 24°C in glass flasks for one week. Logarithmic growth-phase cells (OD_730_ ≃ 0.3) were harvested by centrifugation (7,000 × *g*, 10 min, 4°C). The pelleted cells were suspended in 3% (w/v) NaCl and centrifuged again (9,000 × *g*, 15 min, 4°C). The pelleted cells were stored at –30°C immediately after freezing them with liquid N_2_.

### Chemicals

Chemicals and reagents were obtained from Wako Pure Chemicals (Osaka, Japan) or Nacalai Tesque (Kyoto, Japan) unless otherwise noted. Synthetic peptide substrates were obtained from the Peptide Institute (Osaka, Japan). Molecular mass markers were purchased from Sigma-Aldrich (Tokyo, Japan) and Bio-Rad (Tokyo, Japan). Toyopearl Butyl-650M and Toyopearl DEAE-650S were obtained from Tosoh (Tokyo, Japan).

### Growth curve and chlorophyll *a* assay

Equivalent numbers of *Symbiodinium* sp. KB8 cells were inoculated into 100 ml of f/2 medium. The OD_730_, as the measure of cell proliferation, chlorophyll *a* concentration, and protease activity (see below) were measured once a week. The chlorophyll *a* concentration in a 90% (v/v) acetone extract was calculated as described by Jeffrey and Humphrey [13].

### CP assay

A slightly modified version of a published CP assay [11, 14] utilized Boc-VLK-MCA as the substrate. Briefly, each reaction contained 50 μl of 100 mM succinate-borate (pH 4.0), 10 μl of 10 mM tris(2-carboxyethyl) phosphine hydrochloride, 29 μl distilled water, and 1 μl of 10 mM Boc-VLK-MCA dissolved in DMSO. The reaction was initiated by adding 10 μl of an enzyme solution into 100 μl of the reaction mixture at 37°C. After a 30-min incubation period, the reaction was terminated by adding 2 ml of 1% (w/v) SDS in 100 mM sodium borate (pH 9.0). The fluorescence of the mixture was measured with an F-2500 fluorescence spectrophotometer (Hitachi High-Technologies, Tokyo, Japan; emission wavelength, 460 nm; excitation wavelength, 360 nm). The specific activity was expressed as the amount (mg) of purified enzyme that catalyzed the production of 1.0 μmol of 7-amino-4-methylcoumarin per hour at 37°C. The concentration of this product was determined by comparing its fluorescence intensity to a standard curve using authentic 7-amino-4-methylcoumarin. The synthetic fluorogenic peptides used in this study are listed in S1 Table.

### Purification of VLKP

**Fig 1:**
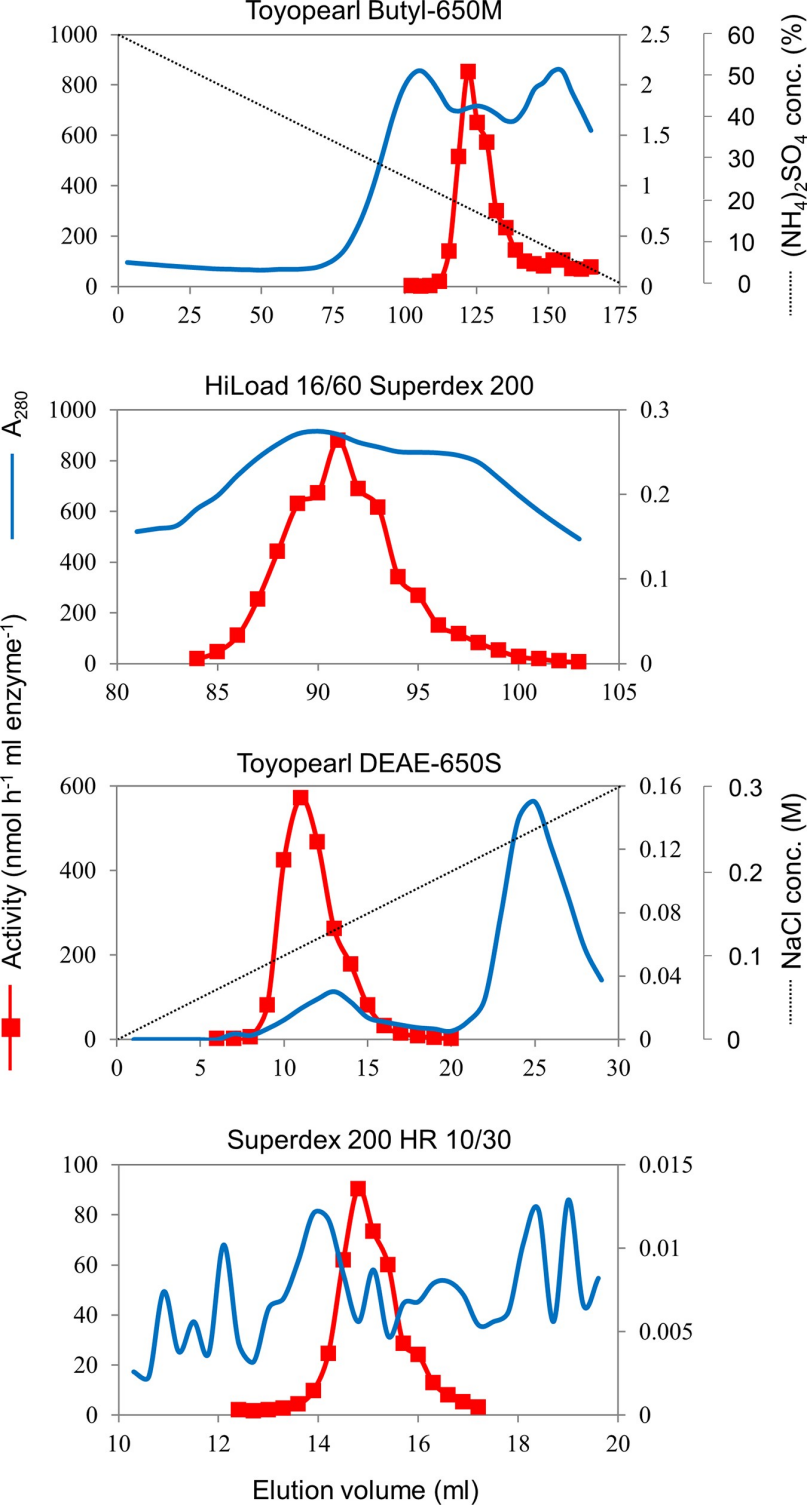
Chromatographic purification of VLKP. Toyopearl Butyl-650M chromatography used a 60–0% (w/v) ammonium sulfate gradient. Toyopearl DEAE-650S chromatography used a 0–0.3 M NaCl gradient. The chromatography sequence is shown top to bottom in the figure.

All enzyme purification procedures were carried out at 4°C. Algal cells were suspended in 20 mM Tris-HCl (pH 8.0) and disrupted by ultrasonication at 140 W (Branson Sonifier 250D, Emerson, Japan). Unbroken cells and debris were removed by centrifugation (17,000 × *g*, 30 min) and then ultracentrifugation (150,000 × *g*, 60 min). The supernatant was filtered through a Miracloth (Calbiochem, Darmstadt, Germany) and the filtrate retained, which was then subjected to a 60% (w/v) (NH_4_)_2_SO_4_ precipitation with stirring for 30 min. The mixture was held for 1 h, after which it was centrifuged (17,000 × *g*, 15 min). Next, the supernatant was applied to a column (2.6 × 15 cm) of Toyopearl Butyl-650M equilibrated with 20 mM Tris-HCl (pH 8.0), 60% (w/v) (NH_4_)_2_SO_4_. The column was washed with three column volumes of the equilibration solution and eluted with 10 column volumes of a 60–0% (NH_4_)_2_SO_4_ linear gradient in 20 mM Tris-HCl (pH 8.0). The fractions with the greatest protease activity (~16% (w/v) (NH_4_)_2_SO_4_) were concentrated in a Centriplus YM-30 apparatus (Millipore, Bedford, MA). The concentrated sample was loaded onto a HiLoad 16/60 Superdex 200 column (GE Healthcare, Tokyo, Japan) equilibrated with 20 mM Tris-HCl (pH 8.0), 0.15 M NaCl, and connected to a ÄKTAprime chromatography system (GE Healthcare). NaCl was included in the eluent to reduce protein adsorption to the surface of the resin. Proteins were eluted in the same solvent at a flow rate of 1.0 ml min^–1^. The fractions with substantial protease activity (eluted at ~91 ml) were pooled, diluted with two volumes of 20 mM Tris-HCl (pH 8.0), and then applied to a Toyopearl DEAE-650S column (1.0 × 1.0 cm) equilibrated with 20 mM Tris-HCl (pH 8.0). The column was washed with three column volumes of the equilibration buffer and eluted with 10 column volumes of a 0–0.3 M linear gradient of NaCl in 20 mM Tris-HCl (pH 8.0). Fractions with substantial protease activity (eluting at ~0.11 M NaCl) were pooled and concentrated in a Minicon CS15 concentrator (Millipore). The concentrated sample was loaded onto a Superdex 200 HR 10/30 column (GE Healthcare) equilibrated with 20 mM Tris-HCl (pH 8.0), 0.15 M NaCl, which was connected to the ÄKTAprime system. Proteins were eluted with the equilibration buffer at a flow rate of 0.3 ml min^–1^. The fractions with substantial protease activity (eluting at ~15 ml) were pooled and dialyzed against 20 mM Tris-HCl (pH 8.0) prior to characterization studies. Typical chromatographic profiles of the purification procedure are shown in Fig 1.

### Measurement of protein concentration

Protein concentration was measured using Pierce BCA Protein Assay kit reagents (Thermo Fisher Scientific, Yokohama, Japan). BSA served as the standard. The protein concentration of each column chromatography fraction was expressed as its OD_280_. Absorbance was measured using a UV-2450 UV-VIS spectrophotometer (Shimadzu, Kyoto, Japan).

### Gel filtration for molecular mass determination

The molecular mass of purified VLKP was estimated by HiLoad 16/60 Superdex 200 gel filtration with the chromatography controlled by the ÄKTAprime system. The column was equilibrated in 20 mM Tris-HCl (pH 8.0), 0.15 M NaCl and the protein eluted in the same buffer at a flow rate of 1.0 ml min^–1^. Fractions of 0.5 ml were collected. The molecular mass markers (Sigma-Aldrich) blue dextran (2,000 kDa), thyroglobulin (669 kDa), alcohol dehydrogenase (150 kDa), BSA (66 kDa), and carbonic anhydrase (29 kDa) were used to calibrate the column.

### SDS-PAGE

Laemmli SDS-PAGE [15] was performed using polyacrylamide gels containing 12 or 14% (w/v) acrylamide. Samples were heated at 95°C in the presence of 2-mercaptoethanol. The gels were stained with silver or Coomassie Brilliant Blue R-250 (CBB). Precision Plus Protein Kaleidoscope Prestained Protein Standards (Bio-Rad) were used to calibrate the gels.

### In-gel digestion of VLKP

A TCA solution (100%, w/v; 250 μl) was added to 1 ml purified VLKP. The sample was incubated for 10 min at 4°C and then centrifuged (15,000 × *g*, 10 min, 4°C). The precipitate was washed twice with 200 μl acetone and centrifuged again (15,000 × *g*, 10 min, 4°C). After removing the acetone, the precipitate was held on ice for 3 min. Then, the sample was suspended in 30 μl SDS-PAGE sample buffer, electrophoresed through an SDS-PAGE gel (12% (w/v) acrylamide), and then silver stained. The predicted VLKP bands were cut into small pieces to increase the surface area and put into a 1.5 ml tube. They were shaken with 100 μl of 15 mM potassium ferricyanide, 50 mM sodium thiosulfate for 10 min and then with 500 μl water for 15 min. The destaining procedure was repeated a second time.

The proteins in the gel pieces were digested with trypsin as described [16]. The gel pieces were washed one time with 50 mM ammonium bicarbonate and then three times with wash buffer containing 50 mM ammonium bicarbonate and 50% (v/v) acetonitrile for 15 min (each time) with vortexing. Acetonitrile (100 μl) was added to the tube to cover the gel pieces completely, with subsequent incubation for 5 min. The gel pieces were then dried completely using a centrifugal concentrator CC-105 (Tomy, Tokyo, Japan). Reduction of cysteine residues was carried out with a 10 mM dithiothreitol (DTT) solution in 50 mM ammonium bicarbonate for 45 min at 56°C. After discarding the DTT solution, the same volume of a 55 mM iodoacetamide solution in 50 mM ammonium bicarbonate buffer was added and incubated in darkness for 30 min at room temperature to achieve alkylation of cysteine residues. The iodoacetamide solution was replaced with wash buffer, and the mixture was vortexed two times for 15 min each. Gel pieces were washed and dried in 100% acetonitrile followed by final drying in a centrifugal concentrator (CC-105).

The dried gel pieces were swollen with 2 μl trypsin solution (10 ng μl^–1^) (Promega, Tokyo, Japan) reconstituted with 50 mM ammonium bicarbonate. The gel pieces were incubated overnight at 37°C. The supernatant was transferred to a new 0.5 ml tube, and the peptides were extracted with 10 μl of 5% formic acid/50% acetonitrile for 10 min in a sonication bath. This step was repeated twice. Samples in extraction buffer were pooled in 0.5 ml tubes and evaporated in a CC-105 centrifugal concentrator. The volume was reduced to approximately 5 μl, and then 10 μl of 0.3% formic acid was added for nano-LC-ESI-MS/MS analysis.

### Nano-LC-electrospray ionization-MS/MS of VLKP tryptic peptides

MS and tandem-MS spectra were obtained using a LC-ESI-LIT-q-TOF spectrometer (NanoFrontier eLD, Hitachi High-technologies) as described [16]. Linear ion trap-time of flight (LIT-TOF) and collision induced dissociation (CID) modes were used for MS detection and peptide fragmentation. The trypsin-treated liquid sample (10 μl) was diluted with formic acid solution to give a final concentration of 0.3% formic acid and then injected. Peptides were trapped with a C18 column (Monolith Trap C18-50-150, Hitachi High-Technologies). Peptide separation was achieved using a packed nano-capillary column (NTCC-360/75-3, Nikkyo Technos, Tokyo, Japan) at a flow rate of 200 nl min^–1^. The separated peptides were then ionized with a capillary voltage of 1700 V. The ionized peptides were detected using a detector potential TOF range of 2050–2150 V. The peptides in the column were eluted using a stepwise acetonitrile gradient (buffer A: 2% acetonitrile, 0.1% formic acid; buffer B: 98% acetonitrile, 0.1% formic acid, gradient protocol: 0 min A = 98%, B = 2%; 60 min A = 60%, B = 40%).

*De novo* sequencing and protein identification was performed using PEAKS Studio software (Bioinformatics Solutions Inc., Waterloo, Canada). A *Symbiodinium* sp. KB8 database was constructed in-house using expressed sequence tag sequences found at http://medinalab.org/zoox/kb8_assembly.fasta.bz2 [17]. *De novo* sequencing was performed using the Peaks algorithm with the following parameters: precursor-ion error tolerance, 0.05 Da; product-ion error tolerance, 0.05 Da; digestion enzyme, trypsin; fixed modifications, cysteine carboxyamidomethylation; variable modifications, histidine, tryptophan and/or methionine oxidation. The sequencing data were subjected to a SPIDER homology search/Blast (PEAKS Studio) against the in-house *Symbiodinium* database.

### Cloning of VLKP cDNA and construction of a vector for rVLKP expression

Frozen *Symbiodinium* sp. KB8 cells were ground to a fine powder under liquid nitrogen using a mortar and pestle. Total RNA in the frozen powder was purified with RNeasy Mini kit reagents (Qiagen, Tokyo, Japan). cDNA was synthesized with PrimeScript 1st strand cDNA Synthesis kit reagents (Takara, Shiga, Japan) and total RNA as the template. To obtain the first half of the VLKP gene, the forward primer was 5'-ATGAACGCGGCCACGGCCTTTG-3' and the reverse primer was 5'-TCAAATAACGATGGCTGTCTCTTCAGCC-3'. RT-PCR was carried out using KOD-Plus-Neo DNA polymerase (Toyobo, Osaka, Japan) and the PrimeScript-synthesized cDNA as the template. Using In-Fusion HD Cloning kit reagents (Takara), the reverse-transcribed amplicons were inserted into a *Sma*I-digested pQE-32 vector downstream of an encoded, in-frame, N-terminal His_6_ tag (Qiagen). Sequencing of the plasmid confirmed correct insertion of the amplicon.

### Expression, purification, and refolding of rVLKP

Expression of the gene encoding VLKP was induced by incubation of *E*. *coli* with with isopropyl β-d-1-thiogalactopyranoside (final concentration, 0.4 mM) for 3 h at 25°C in 2× YT medium containing 50 μg ml^–1^ ampicillin after the cell culture medium had an OD_660_ ~0.5 (culture temperature, 25°C). The harvested cells were suspended in 20 mM Tris-HCl (pH 8.0), 0.5 M NaCl, 5 mM imidazole, 1 mM DTT and disrupted by ultrasonication at 80 W. After centrifugation (20,000 × *g*, 20 min, 4°C), the pellet was suspended in 10 ml denaturation buffer (8 M urea, 20 mM Tris-HCl pH 8.0, 0.5 M NaCl, 5 mM imidazole, 1 mM DTT) and stirred for 30 min at room temperature. After centrifugation (20,000 × *g*, 20 min, 4°C), the pellet was discarded. Ni-NTA agarose resin (1 ml; Qiagen) was added to the supernatant, and the mixture was gently stirred for 2 h at 4°C. Then, the suspension was loaded onto an Econo-Pac disposable chromatography column (Bio-Rad). The column was washed with a 100-ml linear gradient starting with 100% denaturation buffer and ending with 100% wash buffer (3 M urea, 20 mM Tris-HCl (pH 8.0), 0.5 M NaCl, 50 mM imidazole), after which the column was washed with 10 ml wash buffer. The recombinant protein was then eluted three times with a 2-ml volume of 3 M urea, 20 mM Tris-HCl (pH 8.0), 0.5 M NaCl, 1 M imidazole. Proteins in the three elution volumes were separately subjected to SDS-PAGE, and the first wash, which contained the most protein, was dialyzed for 30 min twice against 500 ml of 2 M urea, 20 mM Tris-HCl (pH 8.0), 0.5 M NaCl, 0.5 M imidazole, 10 mM EDTA (ethylenediamine-*N*,*N*,*N*',*N*'-tetraacetic acid), 0.1% (w/v) glycine, then twice against 500 ml of 1 M urea, 20 mM Tris-HCl (pH 8.0), 0.5 M NaCl, 10 mM EDTA, 0.1% (w/v) glycine, and finally twice against 500 ml of 137 mM NaCl, 2.7 mM KCl, 10 mM Na_2_HPO_4_, 1.76 mM KH_2_PO_4_, 1% (w/v) glycine (pH 7.4) lacking urea.

### Western blotting

A monoclonal antibody against polyHis (Clone HIS-1, Sigma-Aldrich) served as the primary antibody, and alkaline phosphatase–conjugated anti-mouse IgG (Cat#A3562, Sigma-Aldrich) served as the secondary antibody. Nitro-blue tetrazolium chloride (Wako Pure Chemicals) and 5-bromo-4-chloro-3-indolyphosphate *p*-toluidine (Wako Pure Chemicals) were used for visualization.

## Results

### Protease activity in the crude extract of *Symbiodinium* cells

The protease activity of the crude extract of *Symbiodinium* sp. KB8 was found to be maximum for Boc-VLK-MCA among the six synthetic fluorogenic peptide substrates (S1 Fig). The activity for each substrate was greatest between pH 4 and 4.5. To determine the relationship between protease activity and cell proliferation, VLKP activity, chlorophyll *a* concentration, and OD_730_ were measured at each *Symbiodinium* growth phase (S2 Fig). Activity during the late logarithmic growth phase was greater than during other phases. Therefore, to purify and characterize VLKP, cells were harvested during late logarithmic growth (OD_730_ ⋍ 0.3).

### Purification of VLKP

After ammonium sulfate fractionation, four chromatography steps were sufficient to purify VLKP to near homogeneity. The ammonium sulfate–fractionated extract from *Symbiodinium* sp. KB8 cells (wet weight, 31.4 g) was sequentially chromatographed through columns of Toyopearl Butyl-650M, HiLoad 16/60 Superdex 200, Toyopearl DEAE-650S, and Superdex 200 HR 10/30. After Toyopearl DEAE-650S chromatography, although the specific activity of the enzyme fraction had increased 566-fold in comparison with that of the crude extract, upon Superdex 200 HR 10/30 chromatography which followed, the specific activity of the sample decreased to 156-fold that of the activity in the crude extract, and the yield was a meager 0.4%. This yield loss could be partly attributed to denaturation and adhesion of the protease to the Minicon filtration apparatus just prior to Superdex 200 chromatography. Although the specific activity decreased during Superdex 200 chromatography, this step was retained for final purification because it eliminated most of the remaining contaminating proteins (refer to the protein profile in Fig 1) and thus allowed us to carry out a definitive peptide sequence analysis (see below). A summary of the purification procedure is presented in Table 1, and typical chromatograms are presented in Fig 1. Judging from the results of the SDS-PAGE gel of the purified sample, the CP activity was associated with two bands (S3 Fig). Comparison of the activity associated with protein fractions from the Superdex 200 chromatography and their positions in the SDS-PAGE gel indicated that the upper band (31.3 kDa) was VLKP. LC-MS/MS revealed that the lower band (26 kDa) was Fe-superoxide dismutase, which we presumed was not involved in VLKP activity.

**Table 1:** VLKP purification, with Boc-VLK-MCA serving as the substrate.

Purification step	Total protein (mg)	Total activity (nmol h^–1^)	Specific activity (nmol h^–1^ mg protein^–1^)	Purification (fold)	Yield (%)
Crude extract	2777	31891	11.48	1.00	100
(NH_4_)_2_SO_4_ fractionation	2082	31530	15.14	1.32	99
Toyopearl Butyl-650M	227.5	13620	59.87	5.21	42
HiLoad 16/60 Superdex 200	8.48	5441	641.8	55.9	17
Toyopearl DEAE-650S	0.33	2139	6502	566	6.7
Superdex 200 HR 10/30	0.07	125	1791	156	0.39

### VLKP is monomeric and most active at low pH

The molecular mass of purified, native VLKP was estimated by Superdex 200 chromatography (S4 Fig). The VLKP elution volume (92.5 ml) in comparison with that of each of the molecular mass markers indicated that its molecular mass is 31.7 kDa. When denatured by 2% SDS and boiling, according to its position in an SDS-PAGE gel, its molecular mass is 31.3 kDa. The fact that these two molecular mass values agree indicated that VLKP is a monomer.

The pH optimum for purified VLKP activity was examined in 0.1 M succinate-borate solutions with pH values between 3.0 and 5.0 and in 0.1 M MES-HEPES-Tricine solutions with pH values between 5.0 and 6.0. Maximum activity was found between pH 4.0 and 4.5; above pH 5, ~90% of the activity was lost (S5 Fig). These activity values were consistent with those observed with the substrate BOK-VLK-MCA using crude extract of *Symbiodinium* sp. KB8 (S1 Fig). The fact that maximum activity occurred at relatively low pH values suggested that VLKP may function in acidic organelles.

Substrate specificity of purified VLKP was examined using the synthetic fluorogenic peptides at pH 4.0 (Table 2). As found for the crude extract, the purified enzyme was most active with Boc-VLK-MCA.

**Table 2:** Substrate specificity of purified VLKP.

Substrate	Relative activity (%)
Ac-DEVD-MCA	5.3 ± 0.7
Ac-YVAD-MCA	11.1 ± 0.7
Boc-LRR-MCA	6.4 ± 1.0
Boc-VLK-MCA	100.0 ± 4.9
Suc-LLVY-MCA	14.2 ± 0.3
Z-LLE-MCA	52.0 ± 0.9

Relative activity for Boc-VLK-MCA was set to 100%, and the activity for each of the other substrates was scaled to that value. Synthetic fluorogenic peptides were added to a final concentration of 0.1 mM. Values represent the mean ± SE of three independent experiments.

### Effects of heat treatment and protease inhibitors on VLKP activity

The optimum temperature and thermal stability of purified VLKP were determined (S6 Fig). Although the optimum growth temperature of *Symbiodinium* sp. KB8 was found to be 24°C, VLKP was most active at 40°C (S6(A) Fig). However, the stability of VLKP decreased as the temperature increased, with the temperature effect being especially noticeable above 30°C (S6(B) Fig). Only 50% of the activity remained at 40°C, and 10% remained at 60°C. The stability of VLKP was measured by holding the enzyme in the absence of substrate at the designated temperature for 10 min and then measuring the residual activity under standard conditions at 37°C, whereas the experiments relating activity and temperature were performed at the designated temperature in the presence of substrate. The differences in the curves shown in S6 Fig. suggested that the protease-substrate complex is more stable than the protease itself.

To determine the type of VLKP active site, the effects of various protease inhibitors on its activity were tested (Table 3). Proteases can be classified into four groups, namely aspartic protease, CP, serine protease, and metalloprotease. These proteases have an oxyanion hole which make their substrate susceptible to attack by a nucleophile [18]. Activity significantly decreased in the presence of CP inhibitors, i.e., 10 μM leupeptin (98% inhibition), 1 μM trans-epoxysuccinyl-l-leucyl-amido(4-guanidino)butane (98% inhibition), 1 mM antipain (94% inhibition), or 10 mM *N*-ethylmaleimide (90% inhibition). In addition, serine protease inhibitors also affected activity, i.e., 1 mM PMSF (65% inhibition) or 1 mM *N*-tosyl-l-phenylalanine chloromethylketone (97% inhibition), but these inhibitors were similar or less potent than were the CP inhibitors. Conversely, EDTA and EGTA (*O*,*O*'-bis(2-aminoethyl)ethyleneglycol-*N*,*N*,*N*',*N*'-tetraacetic acid) (each of which inhibits metalloproteases) or pepstatin A (inhibits aspartic proteases) did not affect activity. These results indicated that VLKP is either a CP or serine protease.

**Table 3:** Effects of protease inhibitors on the VLKP activity.

Inhibitor	Target protease	Concentration (mM)	Relative activity (%)
None			100.0 ± 1.3
Pepstatin A	Asp	0.1	63.1 ± 4.4
Antipain	Cys	1	5.7 ± 0.3
		0.1	5.6 ± 0.5
		0.01	9.0 ± 0.4
E-64	Cys	0.1	2.8 ± 0.1
		0.01	2.8 ± 0.2
		0.001	2.0 ± 1.1
NEM	Cys	10	12.9 ± 2.2
		1	38.8 ± 1.8
		0.1	70.4 ± 0.8
Leupeptin	Cys and Ser	1	3.0 ± 1.0
		0.1	2.8 ± 0.2
		0.01	2.0 ± 1.0
PMSF	Ser	1	35.1 ± 2.6
		0.1	61.3 ± 4.4
TPCK	Ser	1	3.0 ± 1.1
		0.1	6.0 ± 0.2
		0.01	17.7 ± 2.4
EDTA	Metal	1	67.8 ± 9.0
		0.1	63.6 ± 7.2
EGTA	Metal	1	88.2 ± 5.7
		0.1	85.6 ± 5.7

Addition of distilled water to a reaction served as the control (None). Activity for the control was set to 100%, and activities for the other conditions were scaled to that value. Values represent the mean ± SE of three independent experiments. Inhibitor: E-64, trans-epoxysuccinyl-l-leucyl-amido(4-guanidino)butane; NEM, *N*-ethylmaleimide; PMSF, phenylmethylsulfonyl fluoride; TPCK, *N*-tosyl-l-phenylalanine chloromethylketone; EDTA, ethylenediamine-*N*,*N*,*N*',*N*'-tetraacetic acid; EGTA, *O*,*O*'-bis(2-aminoethyl)ethyleneglycol-*N*,*N*,*N*',*N*'-tetraacetic acid. Target protease: Asp, aspartic protease; Cys, cysteine protease; Ser, serine protease; Metal, metalloprotease.

### VLKP is a CP

The cysteine thiol of a CP can be oxidized via nucleophilic attack by a reactive oxygen species, leading to loss of activity. Conversely, CP activity increases in the presence of thiol-reducing agents [19], which prevent oxidation of the active-site cysteine. We, therefore, tested the effects of thiol-reducing agents on VLKP activity (Table 4) and found that it was significantly increased in the presence of 10 mM DTT (10.9-fold) or 10 mM tris(2-carboxyethyl) phosphine hydrochloride (TCEP-HCl) (8.6-fold), but activity was less affected by 10 mM 2-mercaptoethanol (2-ME) or reduced glutathione (GSH) (each >2.5-fold) and hardly affected by cysteine (>1.5-fold), similar to published results [19]. These results indicated that VLKP is a CP.

**Table 4:** Effects of thiol-reducing agents on VLKP activity.

Reducing agent	Concentration (mM)	Relative activity (%)
None		100.0 ± 01.3
2-ME	10	282.7 ± 07.0
	1	82.3 ± 04.3
	0.1	74.9 ± 11.3
Cysteine	10	153.2 ± 01.3
	1	105.5 ± 00.5
	0.1	112.0 ± 03.4
DTT	10	1087.0 ± 05.3
	1	751.2 ± 22.2
	0.1	319.2 ± 14.9
GSH	10	382.0 ± 10.3
	1	138.3 ± 05.8
	0.1	77.2 ± 03.2
TCEP-HCl	10	875.4 ± 23.5
	1	739.3 ± 23.3
	0.1	298.0 ± 17.8

Addition of distilled water to a reaction served as the control (None). Activity for the control was set to 100%, and activities for the other conditions were scaled to that value. Values represent the mean ± SE of three independent experiments. 2-ME, 2-mercaptoethanol; DTT, dithiothreitol; GSH, reduced glutathione; TCEP-HCl, tris(2-carboxyethyl) phosphine hydrochloride.

The possible effects of various metal ions on VLKP activity were examined (1 mM each; S2 Table). The presence of Mg^2+^ or Fe^2+^ had a relatively small but noticeable effect on activity (>1.5-fold). However, in the presence of a chelating agent, e.g., EDTA or EGTA, activity did not decrease (Table 3), indicating that divalent metal ions are not essential for VLKP activity but may stabilize its structure. In addition, activity was significantly inhibited by CuSO_4_, a powerful oxidant, which in the presence of oxygen, i.e., air, catalyzes disulfide bond formation, also suggesting that VLKP is a CP containing a catalytically important cysteine.

### VLKP is encoded as a tandem repeat pro-peptidase

**Fig 2:**
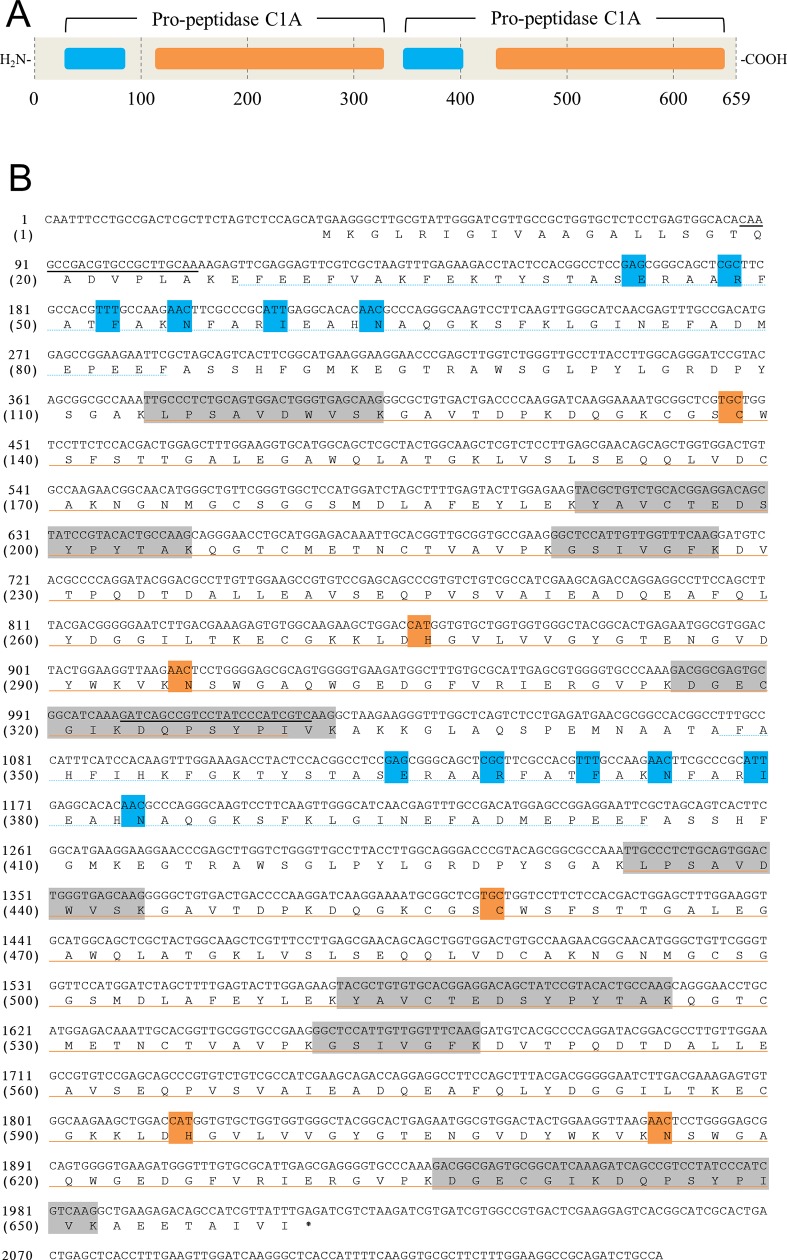
Predicted VLKP sequence. A, Schematic of the predicted precursor VLKP showing its domain structure. Numbers below the schematic are the residue numbers. Blue rectangle, Inhibitor domain; orange rectangle, peptidase domain. B, cDNA sequence (upper) and deduced amino acid sequence (lower) of VLKP. Blue dotted line, Inhibitor domain; orange line, peptidase domain; blue square, ERFNIN motif; orange square, predicted catalytic triad motif; black line, annealing sites of primers for the cDNA encoding rVLKP. The sequences determined by LC-MS/MS are highlighted by gray.

Purified VLKP was digested with trypsin and the resultant peptides subjected to LC-MS/MS sequencing with subsequent analysis with BLAST. The peptide amino-acid sequences were then compared with amino-acid sequences in our in-house *Symbiodinium* expressed sequence tag database. We used part of the VLKP amino acid sequence to construct specific PCR primers to reverse transcribe its gene to yield a cDNA (Fig 2). A search for motif sequences in VLKP classified it as a member of the peptidase C1A superfamily (C1A). C1As are conserved in a variety of organisms, e.g., the plant vacuolar proteases, papain and aleurain, and the animal lysosomal protease, cathepsin. Bacteria, fungi and protists also have C1As [9]. The predicted primary structure of VLKP includes two highly conserved tandemly repeated pro-peptidase sequences (identity of the repeated amino acid sequences: 98%, and DNA sequences: 97%). Specifically, the sequences between Lys^37^ and Ala^332^ and between Lys^357^ and Ala^652^ are identical. Although the precursor is predicted to have a molecular mass of ~71 kDa, the gel filtration and SDS-PAGE results indicated that VLKP is a 31- to 32-kDa monomer. Notably, a termination codon was not found between the tandem sequences. These results indicated that the precursor is likely to be cleaved posttranslationally.

Two domains were apparent within each VLKP pro-peptidase—an inhibitor prodomain and a peptidase domain. The sequence of the inhibitor prodomain is very similar to those of the inhibitor family I29, which are conserved in proC1As. I29 family domains contain the ERFNIN motif (ExxxRxxxFxxNxxxIxxxN), which is also present in the VLKP prodomain (Fig 2). Furthermore, the typical C1A catalytic triad (Cys, His, and Asn) appears to be conserved in the VLKP peptidase domain (Fig 2). Homology modeling with SWISS-MODEL server predicted that VLKP has a 3D structure similar to that of a known C1A [20]. C1As have an oxyanion hole that contains these three residues [18], and VLKP likely has the same system.

### VLKP degrades C1A-specific substrates

To confirm that VLKP can be classified as a C1A and has C1A function, the ability of VLKP to degrade C1A-specific substrates was tested (Table 5). The ability of VLKP to degrade benzyloxycarbonyl-Phe-Arg-4-methylcoumaryl-7-amide (Z-FR-MCA), which only cathepsin B/L degrades, and benzyloxycarbonyl-Leu-Arg-4-methylcoumaryl-7-amide (Z-LR-MCA), which only cathepsin K/S/V and papain degrade, were compared with its ability to degrade Boc-VLK-MCA. For Z-FR-MCA, 152% (in crude extract) and 77% (purified enzyme) of the activity found for Boc-VLK-MCA (100%) was observed; for Z-LR-MCA, the respective values were 140% and 120%. The fact that both peptides were cleaved by purified VLKP supports its categorization as a C1A. That the activity for Z-FR-MCA was less for purified enzyme than for the crude extract suggests that an unidentified C1A(s) closely related to cathepsin B/L might exist in the crude extract and was likely removed during the last step of column chromatography. Furthermore, VLKP is likely to be more closely related to cathepsin K/S/V or papain.

**Table 5:** Crude extract and purified rVLKP specificity for C1A-specific substrates.

Substrate	Target protease	Relative activity (%)	
		Crude extract	Purified enzyme
Boc-VLK-MCA	Plasmin and calpain	100.0 ± 3.4	100.0 ± 5.3
Z-FR-MCA	Cathepsin B/L	152.2 ± 4.8	76.8 ± 8.1
Z-LR-MCA	Cathepsin K/S/V and papain	140.5 ± 6.8	120.3 ± 8.5

Relative activity for Boc-VLK-MCA hydrolysis was set to 100%, and the activity for the other substrates was scaled to that value. Final concentration of each peptide was 0.1 mM. Values represent the mean ± SE of three independent experiments. Z-FR-MCA, benzyloxycarbonyl-Phe-Arg-4-methylcoumaryl-7-amide; Z-LR-MCA, benzyloxycarbonyl-Leu-Arg-4-methylcoumaryl-7-amide.

### Recombinant and native VLKP have similar substrate specificities

**Fig 3:**
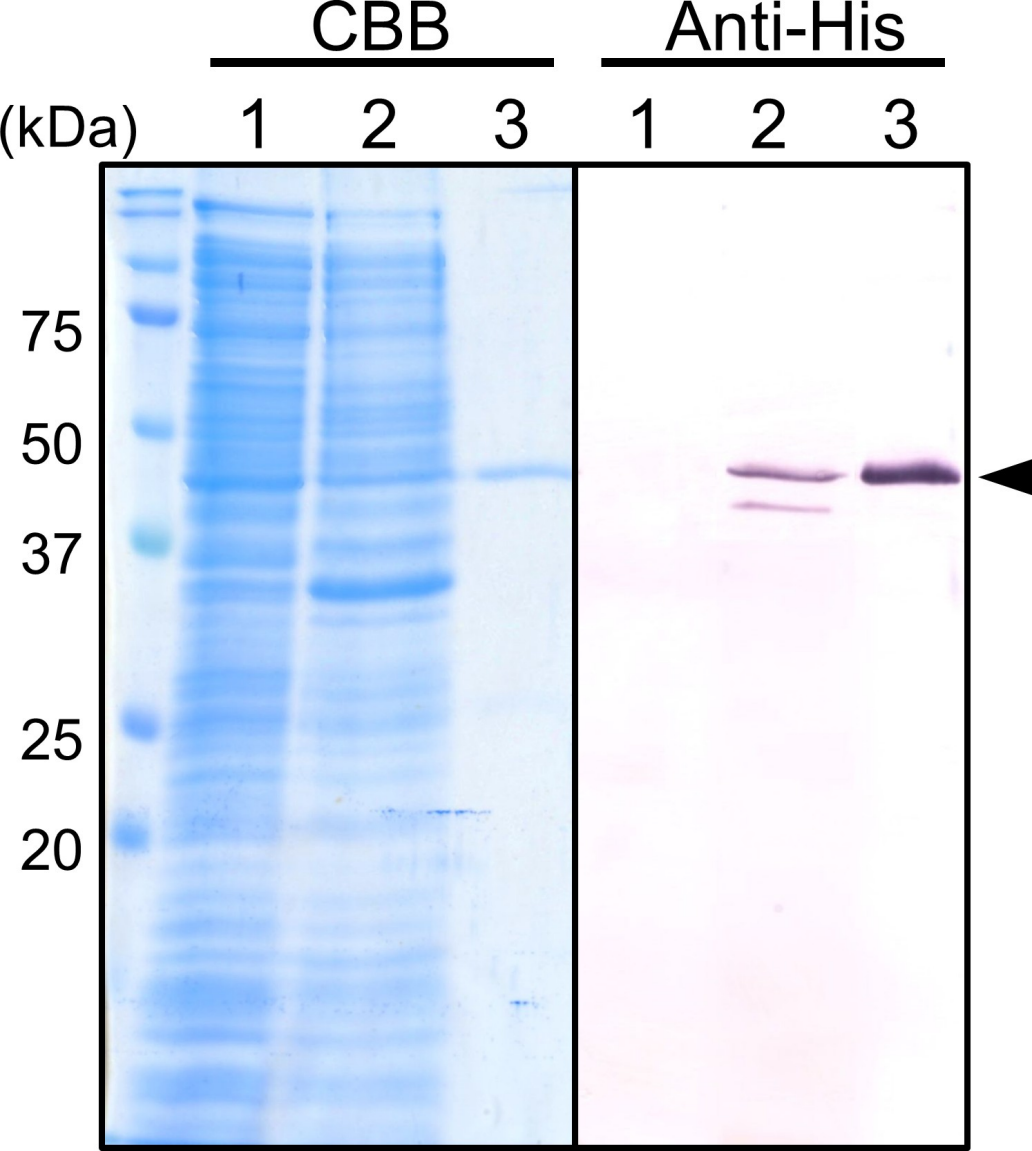
SDS-PAGE and western blotting of rVLKP. rVLKP expressed in *E*. *coli* was subjected to SDS-PAGE followed by CBB staining (left panel, CBB) and western blotting with anti-His tag (right panel, Anti-His). Lane 1, supernatant after centrifugation of lysed *E*. *coli* cells; lane 2, precipitate suspended in denaturation buffer after centrifugation of lysed *E*. *coli* cells; lane 3, rVLKP purified from the precipitate by affinity chromatography.

The N-terminal peptidase domain was expressed in *E*. *coli* as a His-tagged protein (rVLKP) to determine whether the cDNA encodes active VLKP. rVLKP was solubilized and purified by affinity chromatography. A single band for rVLKP was observed upon SDS-PAGE and western blotting (Fig 3). Although the molecular mass of rVLKP was predicted to be 36.8 kDa with the His-tag, the SDS-PAGE band migrated at ~42 kDa. The electrophoretic difference might be caused by the presence of the His-tag. After centrifugation of the sonicated *E*. *coli* cells, the soluble and insoluble/pelleted fractions were compared. Although the CBB-stained gels revealed an intense band of molecular mass ~42 kDa, western blotting for VLKP revealed its presence only in the insoluble fraction. These results suggested that much of expressed rVLKP was in inclusion bodies. Furthermore, for the western-blotted insoluble fraction, a faint band was observed under the rVLKP band (39 kDa, Fig 3). This band was not detected after affinity chromatography (Fig 3), and its identity remained unknown.

We assessed the substrate specificity of purified rVLKP (Table 6). Compared with the activity of native VLKP purified from *Symbiodinium* cells, rVLKP had similar activity. However, Ac-YVAD-MCA, Boc-LRR-MCA, and Suc-LLVY-MCA were less degraded by rVLKP, which may have been a consequence of impurities remaining in the native VLKP sample.

**Table 6:** Comparison of substrate specificity of native and recombinant VLKP.

Substrate	Relative activity (%)	
	Native VLKP	Recombinant VLKP
Boc-VLK-MCA	100.0 ± 4.9	100.0 ± 2.8
Ac-DEVD-MCA	5.3 ± 0.7	4.7 ± 0.3
Ac-YVAD-MCA	11.1 ± 0.7	N.D.
Boc-LRR-MCA	6.4 ± 1.0	N.D.
Suc-LLVY-MCA	14.2 ± 0.3	4.6 ± 1.2
Z-LLE-MCA	52.0 ± 0.9	32.1 ± 2.0
Z-FR-MCA	76.8 ± 8.1	79.3 ± 4.5
Z-LR-MCA	120.3 ± 8.5	125.9 ± 4.8

Relative activity with Boc-VLK-MCA was set to 100%, and the activity for each of the other substrates was scaled to that value. Final concentration of each peptide was 0.1 mM. Values represent the mean ± SE of three independent experiments. Activities for native VLKP are those of Tables 2 and 5. N.D., not detectable.

## Discussion

### Identification and characterization of VLKP

The predicted VLKP amino acid sequence contains two conserved C1A-type prosequences (Fig 2). Based on its predicted sequence, VLKP should have a molecular mass of ~71 kDa; native VLKP, however, was characterized as a monomer of 31–32 kDa (S4 Fig). Because we did not express full-length rVLKP in *E*. *coli*, we could not assess how the tandem repeat is cleaved, i.e., by autolysis or proteolysis by another enzyme(s). Further studies are thus needed to understand the activation mechanism of VLKP.

Precursors of C1As have the inhibitor prodomain, I29, located N-terminally to the peptidase domain. The I29 sequence includes the characteristic motif ERFNIN, and this domain inhibits the activity of the peptidase domain. C1As are activated when the I29 is autocatalytically cleaved upon a decrease in cellular pH [21, 22]. In addition, I29 is needed for the correct folding and membrane anchoring of the precursor [21]. VLKP contains an ERFNIN motif (Fig 2) and is most active at pH 4.0–4.5, but it is hardly active at higher pH values (S5 Fig), which suggests that excision of the inhibitor domain occurs at pH < 5. Furthermore, a catalytic triad motif (Cys, His, Asn) probably exists in the VLKP peptidase domain (Fig 2) [18].

Precursors of papain-like proteases, i.e., C1As found in plant vacuoles, have an I29 domain as well as a Pro-rich prodomain and granulin prodomain that are C-terminal to the peptidase domain [23, 24]; however, cathepsin-like proteases, which are also C1As and found in animal lysosomes, have only an I29 domain. Alveolata, e.g., the ciliate *Philasterides dicentrarchi* and the malaria protozoan *Plasmodium falciparum*, also express C1A precursors in which only an I29 domain is found. A C-terminal prodomain is not present in the VLKP precursor (Fig 2), indicating that VLKP and other Alveolata C1As may be phylogenetically more closely related to those of animals than those of plants. However, our BLAST search revealed that the sequence similarity of VLKP is greater for Alveolata C1As (⋍55% at best) than for those of plants (⋍50%) or of animals (⋍40%). Recently, a new eukaryotic taxonomy based on molecular phylogenetics classified animals into an Amorphea cluster and classified Alveolata and plants into a Diaphoretickes cluster [25]. The greater similarity between Alveolata and plant C1As is consistent with this type of taxonomy. Therefore, Alveolata and plant C1As possibly evolved from a common ancestor, and plant C1As might have acquired their Pro-rich and granulin prodomains after divergence from Alveolata.

### Possible physiological function of VLKP

Plant C1As are involved in pathogen perception, disease-resistance signaling, defense against insects, and senescence [18]. In higher animals, most C1As are mainly involved in intracellular proteolysis or metabolic regulation [26]. Alveolata C1As, especially those of apicomplexa, destroy membrane barriers or immune system components in their hosts so as to obtain nutrition via digestion of host proteins [6]. Because most C1As have an inhibitor domain and are activated under acidic conditions, most plant C1As are located in the acidic environment of vacuoles and animal C1As in the acidic environment of lysosomes.

To date, no information has been available concerning the location(s) and function(s) of *Symbiodinium* C1As. However, VLKP is most active at pH ≤ 4.5 (S5 Fig), suggesting that it resides in acidic organelles, e.g. vacuoles, as do other C1As. In addition, because more VLKP-type activity is found in the late logarithmic phase, VLKP is likely to be involved in late-stage metabolism, e.g., senescence and/or nitrogen recycling (note that the medium used for this study, namely f/2, has a low nitrogen concentration, i.e., <1 μM). In fact, the cathepsin-like C1As are the most abundant proteases present during leaf senescence [27]. Notably, corals receive most of their essential amino acids from their intracellular symbionts [8]; as such, amino acid production by *Symbiodinium* is important to mutualism. We, therefore, predict that VLKP will be found in acidic vacuoles and that VLKP-mediated protein degradation during the late logarithmic phase of *Symbiodinium* should counter the effects of nitrogen deficiency by providing nitrogen from proteins no longer required for survival. In the dinoflagellate *Peridinium gatunense*, a CP may be involved in programmed cell death [28]. However, because this protease seems to function extracellularly at pH > 5 [29], this protease is probably not related to VLKP.

To evaluate the *in vivo* function of VLKP, we need to identify its subcellular location and substrates. *Symbiodinium* morphology, growth rate, and gene-expression pattern differ when it is free living or in a symbiotic relationship. It would be interesting to compare VLKP activity in these two types of cells. Hence, it may be fruitful to compare VLKP activity in these two types of cells.

## Supporting information

S1 Fig Substrate specificity of the crude *Symbiodinium* sp. KB8 extract. A MES + HEPES + Tricine solution (pH 4–8) was used for the measurements. Synthetic fluorogenic peptides were each added to a final concentration of 0.1 mM. Values represent the mean ± SE of three independent experiments.
(PDF)

S2 Fig Time course of protease activity, chlorophyll content, and growth during *Symbiodinium* sp. KB8 culture. Protease activity is reported as the activity per amount of protein in the crude extract. Values represent the mean ± SE of three independent experiments.
(PDF)

S3 Fig SDS-PAGE of fractions isolated by Superdex 200 HR 10/30 chromatography. Ten Superdex 200 HR 10/30 fractions (from elution volume 13.5 ml to 18 ml, see Fig 1) containing substantial activity were electrophoresed through an SDS-PAGE gel and silver stained. The activity of each fraction was also measured. The amount of protein in the upper band (31.3 kDa; red arrow) correlates with VLKP activity (bar chart). LC-MS/MS revealed that the lower band (27.0 kDa, blue arrow) is Fe-superoxide dismutase, which is not likely to be involved in protease activity. Protein molecular mass standards are shown in the left and right lanes.
(PDF)

S4 Fig Molecular mass determination of VLKP by Superdex 200 HiLoad 16/60 gel filtration. Molecular markers used: 1, thyroglobulin (669 kDa); 2, alcohol dehydrogenase (150 kDa); 3, BSA (66 kDa); 4, carbonic anhydrase (29 kDa). *K*_av_ = (*V*_E_−*V*_0_) (*V*_C_−*V*_0_)^–1^; *V*_0_, void volume (ml); *V*_E_, elution volume (ml); *V*_C_, geometric bed volume (ml).
(PDF)

S5 Fig Optimum pH for VLKP activity. Maximum activity was assigned a value of 100%, and the activities in the other samples were scaled to that value. A succinate-borate buffer (0.1 M, SB) was used for pH values between 3.0 and 5.0, and a MES-HEPES-Tricine buffer (0.1 M, MHT) was used for pH values between 5.0 and 6.0. Values represent the mean ± SE of three independent experiments.
(PDF)

S6 Fig Effects of heat treatment on VLKP activity and stability. Maximum activity was assigned a value of 100%, and the activities of the other samples were scaled to that value. Values represent the mean ± SE of three independent experiments. A, Optimum temperature determination. Activity of purified VLKP was measured between 20 and 50°C at 5°C intervals. B, Thermal stability determination. Purified VLKP was held at a temperature between 10 and 60°C for 10 min, and then activity was measured under standard conditions.
(PDF)

S1 Table Synthetic fluorogenic peptides used in this study. The target substrate of each enzyme are described by reference of Peptide Institute (http://www.peptide.co.jp) and MEROPS (http://merops.sanger.ac.uk).
(PDF)

S2 Table Effects of metal ions on the VLKP activity. Each value is shown as a relative value based on the value of the control (distilled water). Each metal ion was at a final concentration of 1 mM. The counterion SO_4_^2–^ used for Cu^2+^ and Fe^2+^, and Cl^−^used for the other cations. Values represent the mean ± SE of three independent experiments. N.D., not detectable.
(PDF)

## Abbreviations

Boc-VLK-MCA: butyloxycarbonyl-Val-Leu-Lys-4-methylcoumaryl-7-amide
C1A: member of the peptidase C1A superfamily
CP: cysteine proteinase
VLKP: Boc-VLK-MCA-degrading cysteine proteinase
