# Supplementary material for: Isolation and characterization of a tandem-repeated cysteine protease from the symbiotic dinoflagellate Symbiodinium sp. KB8
Source: PLoS One. 2019 Jan 31;14(1):e0211534. doi: 10.1371/journal.pone.0211534 (PMC6355014; doi:10.1371/journal.pone.0211534)
Supplement: S1 Fig — A MES + HEPES + Tricine solution (pH 4–8) was used for the measurements. Synthetic fluorogenic peptides were each added to a final concentration of 0.1 mM. Values represent the mean ± SE of three independent experiments. (PDF) [file pone.0211534.s001.pdf]

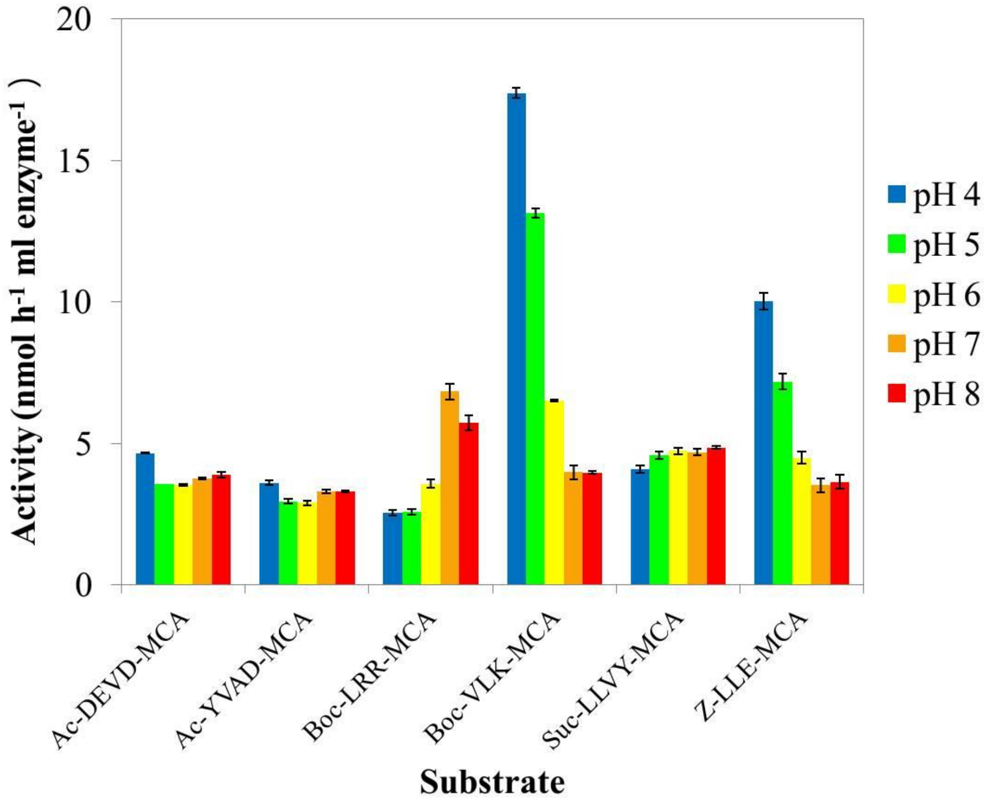

Supplementary FIGURE 1. Substrate specificity of the crude *Symbiodinium* sp. KB8 extract. A MES + HEPES + Tricine solution (pH 4–8) was used for the measurements. Synthetic fluorogenic peptides were each added to a final concentration of 0.1 mM. Values are the mean ± SE of three independent experiments.
