# Supplementary material for: Isolation and characterization of a tandem-repeated cysteine protease from the symbiotic dinoflagellate Symbiodinium sp. KB8
Source: PLoS One. 2019 Jan 31;14(1):e0211534. doi: 10.1371/journal.pone.0211534 (PMC6355014; doi:10.1371/journal.pone.0211534)
Supplement: S2 Fig — Protease activity is reported as the activity per amount of protein in the crude extract. Values represent the mean ± SE of three independent experiments. (PDF) [file pone.0211534.s002.pdf]

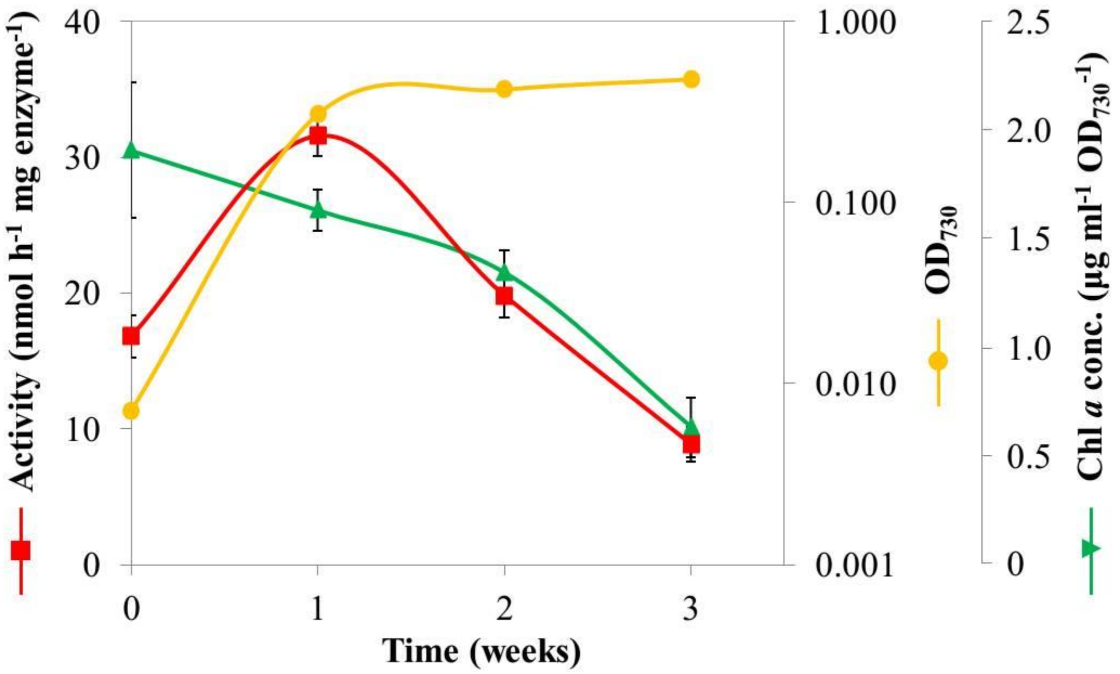

Supplementary FIGURE 2. Time courses of protease activity, chlorophyll content, and growth during *Symbiodinium* sp. KB8 culture. Protease activity is reported as the activity per amount of protein in the crude extract. Values are the mean ± SE of three independent experiments.
