# Supplementary material for: Isolation and characterization of a tandem-repeated cysteine protease from the symbiotic dinoflagellate Symbiodinium sp. KB8
Source: PLoS One. 2019 Jan 31;14(1):e0211534. doi: 10.1371/journal.pone.0211534 (PMC6355014; doi:10.1371/journal.pone.0211534)
Supplement: S3 Fig — Ten Superdex 200 HR 10/30 fractions (from elution volume 13.5 ml to 18 ml, see Fig 1) containing substantial activity were electrophoresed through an SDS-PAGE gel and silver stained. The activity of each fraction was also measured. The amount of protein in the upper band (31.3 kDa; red arrow) correlates with VLKP activity (bar chart). LC-MS/MS revealed that the lower band (27.0 kDa, blue arrow) is Fe-superoxide dismutase, which is not likely to be involved in protease activity. Protein molecular mass standards are shown in the left and right lanes. (PDF) [file pone.0211534.s003.pdf]

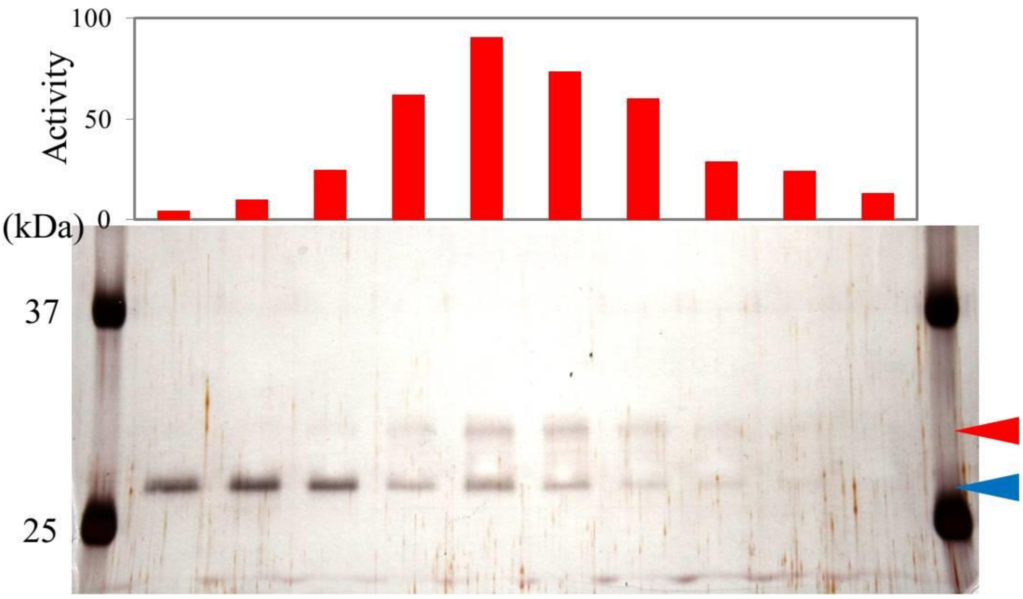


**Supplementary FIGURE 3.** SDS-PAGE of fractions isolated by Superdex 200 HR 10/30 chromatography. Ten Superdex 200 HR 10/30 fractions containing substantial activity were electrophoresed through an SDS-PAGE gel and silver stained. The activity of each fraction was also measured. The amount of protein in the upper band (31.3 kDa; red arrow) correlates with VLKP activity (bar chart). LC-MS/MS revealed that the lower band (27.0 kDa, blue arrow) is Fe-superoxide dismutase, which is not likely to be involved in protease activity. Protein molecular mass standards are shown in the left and right lanes.
