# Supplementary material for: Isolation and characterization of a tandem-repeated cysteine protease from the symbiotic dinoflagellate Symbiodinium sp. KB8
Source: PLoS One. 2019 Jan 31;14(1):e0211534. doi: 10.1371/journal.pone.0211534 (PMC6355014; doi:10.1371/journal.pone.0211534)
Supplement: S4 Fig — Molecular markers used: 1, thyroglobulin (669 kDa); 2, alcohol dehydrogenase (150 kDa); 3, BSA (66 kDa); 4, carbonic anhydrase (29 kDa). Kav = (VE−V0) (VC−V0)–1; V0, void volume (ml); VE, elution volume (ml); VC, geometric bed volume (ml). (PDF) [file pone.0211534.s004.pdf]

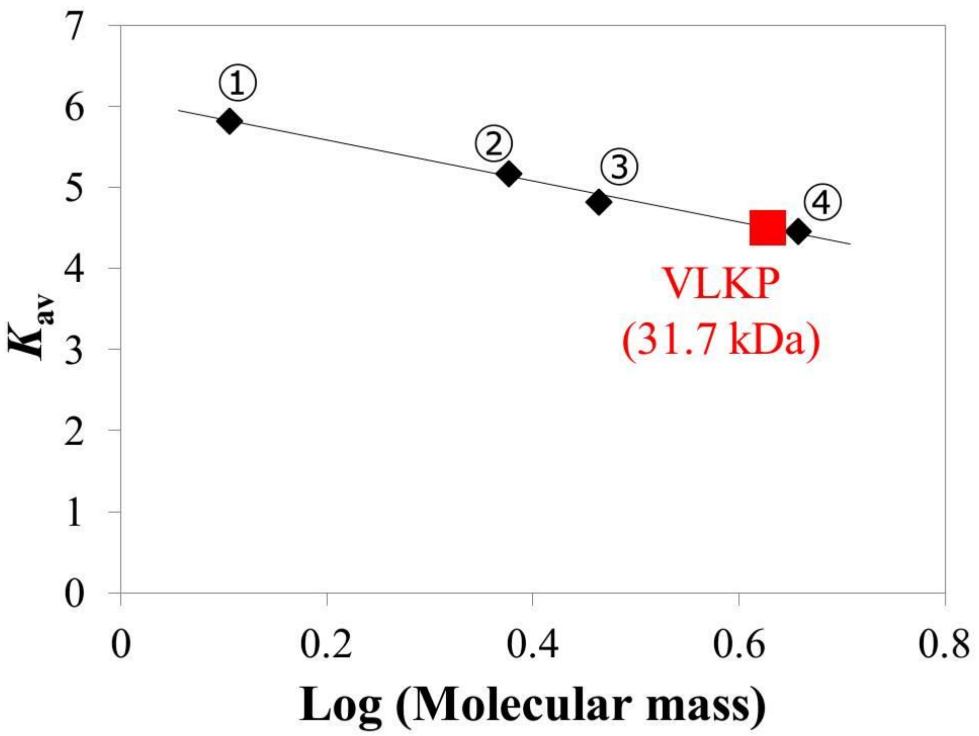


**Supplementary FIGURE 4.** Molecular mass determination of VLKP by Superdex 200 HiLoad 16/60 gel filtration. Molecular markers used: 1, thyroglobulin (669 kDa); 2, alcohol dehydrogenase (150 kDa); 3, BSA (66 kDa); 4, carbonic anhydrase (29 kDa). $K_{av} = (V_E - V_0)(V_C - V_0)^{-1}$; $V_0$, void volume (ml); $V_E$, elution volume (ml); $V_C$, geometric bed volume (ml).
