# Supplementary material for: Isolation and characterization of a tandem-repeated cysteine protease from the symbiotic dinoflagellate Symbiodinium sp. KB8
Source: PLoS One. 2019 Jan 31;14(1):e0211534. doi: 10.1371/journal.pone.0211534 (PMC6355014; doi:10.1371/journal.pone.0211534)
Supplement: S5 Fig — Maximum activity was assigned a value of 100%, and the activities in the other samples were scaled to that value. A succinate-borate buffer (0.1 M, SB) was used for pH values between 3.0 and 5.0, and a MES-HEPES-Tricine buffer (0.1 M, MHT) was used for pH values between 5.0 and 6.0. Values represent the mean ± SE of three independent experiments. (PDF) [file pone.0211534.s005.pdf]

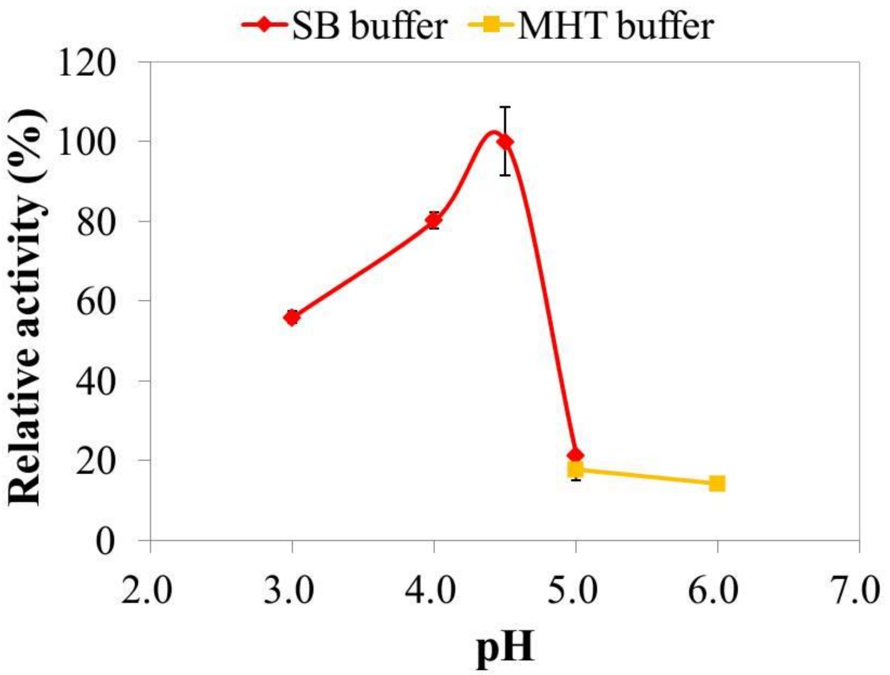

Supplementary FIGURE 5. Optimum pH for VLKP activity. Maximum activity was assigned a value of 100%, and the activities in the other samples were scaled to that value. A succinate-borate buffer (0.1 M, SB) was used for pH values between 3.0 and 5.0, and a MES-HEPES-Tricine buffer (0.1 M, MHT) was used for pH values between 5.0 and 6.0. Values are the mean ± SE of three independent experiments.
