# Supplementary material for: Isolation and characterization of a tandem-repeated cysteine protease from the symbiotic dinoflagellate Symbiodinium sp. KB8
Source: PLoS One. 2019 Jan 31;14(1):e0211534. doi: 10.1371/journal.pone.0211534 (PMC6355014; doi:10.1371/journal.pone.0211534)
Supplement: S6 Fig — Maximum activity was assigned a value of 100%, and the activities of the other samples were scaled to that value. Values represent the mean ± SE of three independent experiments. A, Optimum temperature determination. Activity of purified VLKP was measured between 20 and 50°C at 5°C intervals. B, Thermal stability determination. Purified VLKP was held at a temperature between 10 and 60°C for 10 min, and then activity was measured under standard conditions. (PDF) [file pone.0211534.s006.pdf]

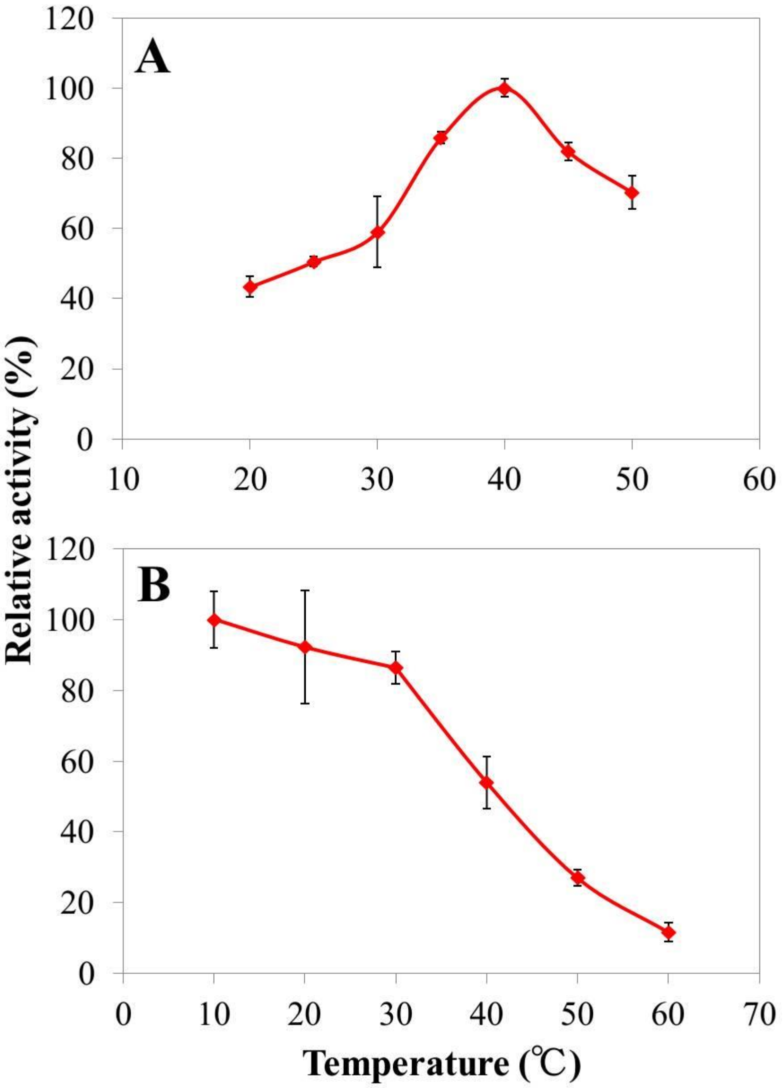

Supplementary FIGURE 6. Effects of heat treatment on VLKP activity and stability. Maximum activity was assigned a value of 100%, and the activities of the other samples were scaled to that value. Values are the mean ± SE of three independent experiments. A, Optimum temperature determination. Activity of purified VLKP was measured between 20 and 50°C at 5°C intervals. B, Thermal stability determination. Purified VLKP was held at a temperature between 10 and 60°C for 10 min, and then activity was measured under standard conditions.
