# Supplementary material for: Isolation and characterization of a tandem-repeated cysteine protease from the symbiotic dinoflagellate Symbiodinium sp. KB8
Source: PLoS One. 2019 Jan 31;14(1):e0211534. doi: 10.1371/journal.pone.0211534 (PMC6355014; doi:10.1371/journal.pone.0211534)
Supplement: S1 Table — The target substrate of each enzyme are described by reference of Peptide Institute (http://www.peptide.co.jp) and MEROPS (http://merops.sanger.ac.uk). (PDF) [file pone.0211534.s007.pdf]

Supplementary TABLE 1. Synthetic fluorogenic peptides used in this study. The target enzymes of each substrate were described by reference of Peptide Institute (http://www.peptide.co.jp) and MEROPS (http://merops.sanger.ac.uk).

Substrate	Preferred target protease	Original name
Ac-DEVD-MCA	Caspase-3/-7/-8	Acetyl-Asp-Glu-Val-Asp-4-methylcoumaryl-7-amide
Ac-YVAD-MCA	Caspase-1	Acetyl-Tyr-Val-Ala-Asp-4-methylcoumaryl-7-amide
Boc-LRR-MCA	Carboxyl side of paired basic residue cleaving enzyme and Proteasome	Butyloxycarbonyl-Leu-Arg-Arg-4-methylcoumaryl-7-amide
Boc-VLK-MCA	Plasmin and Calpain	Butyloxycarbonyl-Val-Leu-Lys-4-methylcoumaryl-7-amide
Suc-LLVY-MCA	Chymotrypsin, Ingensin/Proteasome and Calpain	Succinyl-Leu-Leu-Val-Tyr-4-methylcoumaryl-7-amide
Z-LLE-MCA	Proteasome	Benzyloxycarbonyl-Leu-Leu-Glu-4-methylcoumaryl-7-amide
