# Supplementary material for: Isolation and characterization of a tandem-repeated cysteine protease from the symbiotic dinoflagellate Symbiodinium sp. KB8
Source: PLoS One. 2019 Jan 31;14(1):e0211534. doi: 10.1371/journal.pone.0211534 (PMC6355014; doi:10.1371/journal.pone.0211534)
Supplement: S2 Table — Each value is shown as a relative value based on the value of the control (distilled water). Each metal ion was at a final concentration of 1 mM. The counterion SO42– used for Cu2+ and Fe2+, and Cl−used for the other cations. Values represent the mean ± SE of three independent experiments. N.D., not detectable. (PDF) [file pone.0211534.s008.pdf]

Supplementary TABLE 2. Effects of metal ions on the VLKP activity. Each data is shown as relative value based on the value of the control (distilled water). Each of metal ions was added at the final concentration of 1 mM. Sulfates were used for Cu^{2+} and Fe^{2+}, and chlorides were used for other ions. Values are means ± SE from three independent experiments. N.D., not detected.

Metal ion	Relative activity (%)
None	100.0 ± 4.6
K^{+}	93.1 ± 9.3
Na^{+}	117.0 ± 4.7
Mg^{2+}	157.5 ± 6.7
Mn^{2+}	88.1 ± 7.3
Ni^{2+}	80.5 ± 9.1
Co^{2+}	105.3 ± 5.4
Zn^{2+}	78.6 ± 0.2
Ca^{2+}	128.1 ± 2.0
Fe^{2+}	162.5 ± 3.9
Cu^{2+}	N.D.
